# Motor pathway evaluation by transcranial magnetic stimulation in Swedish horses with acquired equine polyneuropathy

**DOI:** 10.1111/evj.14506

**Published:** 2025-04-21

**Authors:** Anna May, Siv Hanche‐Olsen, Lutz S. Goehring, Kaspar Matiasek, Karin Hultin Jäderlund, Yury Zablotski, Gittan Gröndahl

**Affiliations:** ^1^ Equine Clinic, Centre for Clinical Veterinary Medicine Ludwig Maximilians University Munich Germany; ^2^ Department of Companion Animal Clinical Sciences Norwegian University of Life Sciences Ås Norway; ^3^ Gluck Equine Research Center University of Kentucky Lexington Kentucky USA; ^4^ Section of Clinical & Comparative Neuropathology, Centre for Clinical Veterinary Medicine Ludwig Maximilians University Munich Germany; ^5^ Clinic for Ruminants, Centre for Clinical Veterinary Medicine Ludwig Maximilians University Munich Germany; ^6^ Department of Animal Health and Antimicrobial Strategies Swedish Veterinary Agency Uppsala Sweden

**Keywords:** horse, knuckling, latency time, lower motor neuron, polyneuropathy, Schwannopathy, transcranial magnetic stimulation

## Abstract

**Background:**

Acquired equine polyneuropathy in Nordic horses (AEP) is the most prevalent equine polyneuropathy in Norway, Sweden, and Finland and is characterised by pelvic limb knuckling due to metatarsophalangeal extension dysfunction.

**Objectives:**

To evaluate the function of descending motor pathways in AEP using transcranial magnetic stimulation (TMS).

**Study Design:**

An analytical, observational cohort design.

**Methods:**

Clinical findings and TMS results of 20 horses from an AEP outbreak in Sweden were evaluated at 5‐month intervals. Latency time (LT) in milliseconds (ms) between coil discharge and onset of muscle potential was recorded for thoracic and pelvic limbs.

**Results:**

Fourteen affected horses showed knuckling, 2 horses showed lameness, and 6 horses were neurologically sound and showed no clinical signs at the first visit. Thirteen of 14 neurologically affected horses had improved clinically 5 months later, four no longer showed knuckling. Motor neurological dysfunction with increased LT was confirmed by TMS in all 14 affected horses at both visits. Mean difference in LT from normalised reference values (ΔLT) in the pelvic limbs of affected horses was +12.95 ms (+38%) at the first examination (1.9–29.6 ms; SD 1.23; *n* = 14), and +8.1 ms (+24%) 5 months later (1.0–18.9 ms; SD 1.21; *n* = 14), cutoff >0.8 ms. Eleven of 14 affected horses also presented delayed TMS responses in the thoracic limbs, with up to 14% ΔLT increase. Neurologically sound, non‐lame horses (*n* = 8) showed mean ΔLT −0.5 ms (−1.8 to 0.2 ms; SD = 0.64) in pelvic, and −0.35 ms (range, −0.7 to 0 ms; SD = 0.26; *n* = 8) in thoracic limbs, cutoff >0.2 ms.

**Limitations:**

Examinations were only repeated once.

**Conclusion:**

This study confirms the involvement of motor pathways in AEP and adds to the previously established involvement of sensory nerve fibres. Sensory and motor involvement contributes to the mismatch of ascending and descending nerve signals and to the clinical manifestations. TMS may be useful in evaluating clinical and subclinical cases of AEP.

## 1 INTRODUCTION

Acquired equine polyneuropathy in Nordic horses (AEP) was first reported from Norway in 1995 and has been described in more than 600 cases of different breeds in Sweden, Norway, Finland and recently in Iceland.^1^, ^2^, ^3^, ^4^ The aetiology of the disease is unknown, although a forage‐related cause has remained the main theory.^1^ The characteristic clinical sign of AEP is metatarsophalangeal extension dysfunction (knuckling), that is, an abnormal position with the dorsal side of hooves or fetlocks touching the ground, which mainly occurs in the pelvic limbs. This is associated with peripheral nerve pathology, while affected horses are otherwise bright, alert, and responsive.^1^ Clinical signs vary from mild or intermittent to severe knuckling and recumbency. Besides the knuckling reflecting dysfunctional extension of fetlock joints, AEP has infrequently been associated with stiffness in movements, hypermetric or hypometric strides, but no obvious ataxia.^1^, ^2^, ^3^, ^5^, ^6^ Recumbent horses have a guarded prognosis, and AEP can often be fatal while less severely affected cases have a good long‐term prognosis.^1^, ^7^ Typical histopathology of the nervous system is an inclusion body Schwannopathy with inflammatory changes of the myelin sheath, followed by demyelination and secondary axonopathy.^8^ Nerve fibres in sensory and mixed peripheral nerves, of both thoracic and pelvic limbs, are affected. Examined samples from the central nervous system (CNS—brain, spinal cord) yielded no significant lesions and inconsistent results.^2^, ^5^, ^8^, ^9^ Polyneuropathies can affect various types of nerve fibres, including sensory, motor, and autonomic fibres, either in isolation or in combination, depending on the underlying cause and pathology. The degree of involvement varies depending on the underlying cause, such as diabetic polyneuropathy^10^ or Guillain–Barré Syndrome^11^ in humans. The identification of motor involvement in polyneuropathies is crucial for treatment and reassessment management. In AEP horses, treatment is mainly supportive, but understanding the extent of motor involvement can aid in prognosis and help evaluate recovery times for individual horses. According to previous studies, severely affected horses that survived took significantly longer time to recover compared with mildly affected horses. The median disease duration was ~5 months, though this varied greatly, ranging from 1 day to 2.5 years.^1^, ^7^

Transcranial magnetic stimulation (TMS) is a non‐invasive and painless electrophysiological method in humans and horses to evaluate the descending motor pathways of the nervous system, starting at motor nuclei in the brain all the way down to the muscle.^12^, ^13^, ^14^, ^15^ The method has been used to detect subclinical and clinical abnormalities, to demonstrate the site of a lesion, as well as to monitor neurological disease course and motor recovery in humans.^16^, ^17^ The objective measurement is latency time (LT) in milliseconds (ms) between coil discharge and the onset of muscle potential. Latency time correlates with the length of signal travel distance; hence, LTs of pelvic limbs are longer than those of thoracic limbs. An algorithm developed by Nollet et al. allows normalisation of individual LTs among horses of different sizes by using withers' height.^18^ Any disruption (i.e., conduction block in nerve fibres) in this pathway will cause a delay and therefore a lengthening of LT.^18^, ^19^, ^20^, ^21^ TMS does not distinguish between disruptions from the upper or lower motor neuron system.

The main objectives of this study were twofold: to determine whether (a) clinical AEP increases LTs as an objective indicator for loss or damage of motor pathways integrity, and (b) whether there is a return to normal LTs with clinical improvement over time. Therefore, we measured LTs on two occasions in horses during an active outbreak of AEP.

## 2 MATERIALS AND METHODS

### 2.1 Study population

A selection of cases from an AEP outbreak on premises in Rättvik in mid‐Sweden was studied. The horses belonged to an upper secondary school or were privately owned, and their history was collected from staff, owners, and veterinarians by interviews. The total horse population consisted of 46 horses, of which 2 had arrived during the outbreak. The horses were stabled in individual indoor stalls with daily access to large winter paddocks with a dirt surface. All horses had been fed wrapped forage, one batch until January and another batch from January to March. Confirmation of peripheral neuropathy with myelin sheath degeneration was made by neuropathological examination of the index case in February 2016. During February to April, in total, 24 horses developed clinical signs of AEP, resulting in a prevalence of 52%. As an intervention, the entire stock of wrapped forage (haylage) was exchanged for dry hay by the end of March.

After obtaining informed consent from the owners, a convenience sample of 20 horses (14 of the 24 clinically affected horses and 6 of the 22 non‐affected horses) was included in the study. The study population consisted of 11 Swedish Warmbloods, 6 Swedish Trotters, 2 North Swedish Draught horses, and 1 Irish Sports Horse. There were 13 geldings and 7 mares included. The median age of the horses was 9 years (range 4 to 16 years) and the median withers height was 161.5 cm (range 150–177 cm).

### 2.2 Clinical and neurological examination

All horses in the study population underwent clinical and neurological examination, as well as TMS, and were then divided into three groups: unaffected horses (with no clinical signs), neurologically affected horses, and horses with other clinical signs, including lameness. The horses were examined by the same examiners (2 board‐certified ECEIM diplomates, 1 deputy state officer) twice with 5‐month interval, in May and October 2016. A complete general physical and a full neurologic examination were performed and the horses' withers heights were measured.^22^, ^23^ Horses with an unremarkable neurological examination, and neither knuckling nor a tendency to knuckle, were defined as neurologically sound. Knuckling was defined as an abnormal position with the dorsal side of one or more hooves or fetlocks touching the ground. A tendency to knuckle was defined as incomplete metatarsophalangeal extension involving fetlock instability. The degree of knuckling was graded on the basis of the most severely affected leg, in case of asymmetry, as follows: (0) no knuckling; (1) tendency for knuckling; (2) knuckling but instantaneously replaced in the correct position; (3) knuckling 1–3 s; and (4) knuckling >3 s, according to Gröndahl et al. (Table 1).^1^

**TABLE 1:** Number of horses with different clinical scoring of knuckling, as defined by Gröndahl et al. (2012), in 20 horses evaluated in May and October 2016 at a Swedish premises with cases of AEP.^1^

Score	Clinical definition	May 2016	October 2016
Grade 0	No knuckling	6	10
Grade 1	Tendency for knuckling	4	9
Grade 2	Knuckling but replaced instantaneously	9	1
Grade 3	Knuckling 1–3 s	1	0
Grade 4	Knuckling >3 s	0	0
Total		*20*	*20*

### 2.3 Transcranial magnetic stimulation (TMS)

**FIGURE 1:**
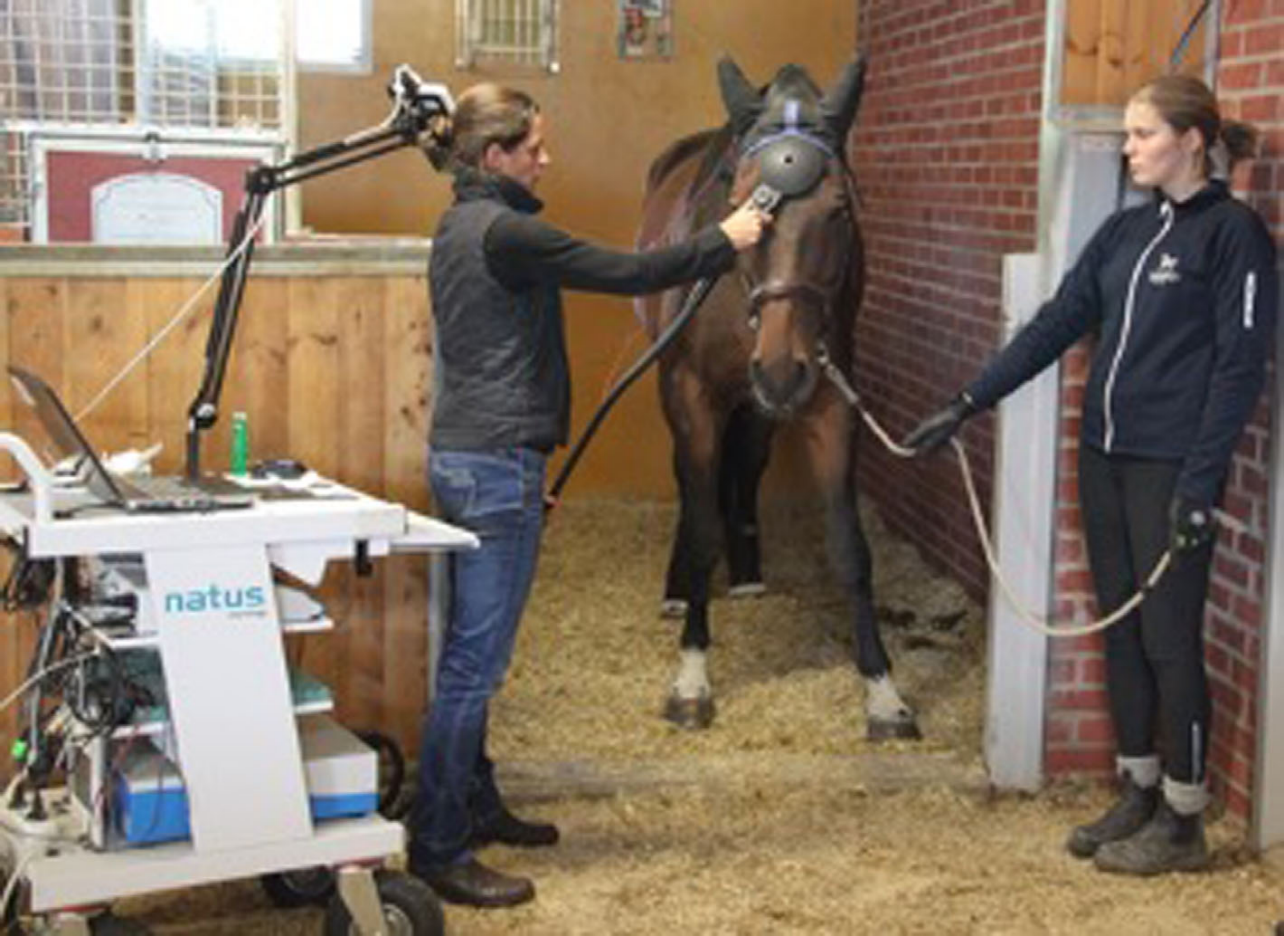
Transcranial magnetic stimulation. Placing of the magnetic coil at the centre of the forehead of the horse. Rättvik, Sweden.

**FIGURE 2:**
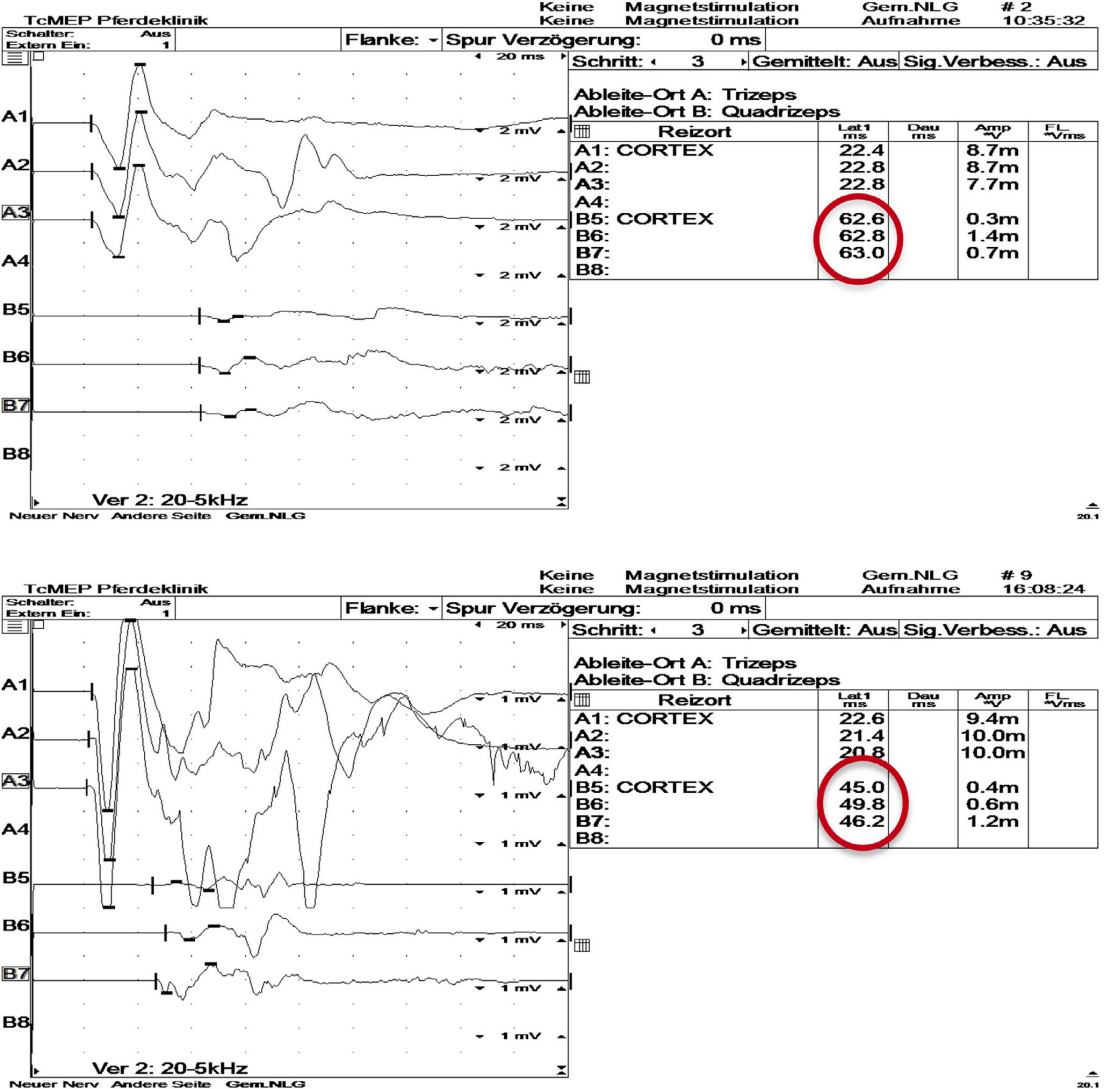
(A, B) Transcranial magnetic stimulation recordings in a horse affected by acquired equine polyneuropathy indicating improvement of neurological impairment. (A) Measurements in May 2016. (B) Measurements in October 2016. On the left, respectively: Graph of magnetic evoked muscle potential in triplicate; A1–A3, thoracic limb; B5–B7, pelvic limb. On the right, respectively: Table with latency times and amplitudes for A1–A3 and B5–B7. Red circles depict latency times for the pelvic limb. Mean latency times calculated from three consecutive measurements: May, thoracic limbs mean 22.7 ms; pelvic limbs mean 62.8 ms; October, thoracic limbs mean 21.6 ms; pelvic limbs mean 47.0 ms.

TMS was performed according to a protocol adapted from Nollet et al. (2004) which had been applied regularly by the authors.^18^, ^19^, ^20^ Horses were sedated with a combination of detomidine (1 mg/100 kg; Cepesedan, CP‐Pharma Handelsgesellschaft mbH) and butorphanol (1 mg/100 kg; Alvegesic, Dechra Veterinary Products Deutschland GmbH). Skin surface EMG electrodes (Natus neurology, disposable adhesive electrodes, Natus Neurology Incorporated) were placed over the right and left extensor carpi radialis (ECR) and tibialis cranialis (CT) muscles (4 measuring points altogether) and reference electrodes were fixed over the humeral lateral epicondyle and the lateral epicondyle of the femur, respectively. The ground electrode was placed over the withers. A circular magnetic coil (MagVenture) generating a magnetic field was then placed on the median of the forehead, as described by Nollet et al. (Figure 1).^18^ The nerve signal elicited by an electromagnetic impulse from the coil placed onto the skin in the skull over the brain descends via upper motor neuron (UMN) tracts in the spinal cord, which activates lower motor neurons (LMNs) with myelinated motor nerve axons in the peripheral nerves. The nerve signal ultimately results in muscle depolarisations, which are measured with electromyography (EMG) via the described skin surface electrodes in the thoracic and pelvic limbs. The latency time (LT) between coil discharge and the onset of muscle potential, measured in milliseconds (ms), was recorded for both thoracic and pelvic limbs (Figure 2). The left and right sides of the horse were examined after each other. The LT was measured three times at 80% intensity (coil output) from each registration site, and the mean value of all measurements of both thoracic limbs and pelvic limbs, respectively, was used. The onset latency times for all horses were compared with reference onset latency times from the literature based on 84 healthy reference horses according to Nollet et al.^21^ This predicted latency time was calculated for each horse based on its wither's height: for ECR muscle, 8.55 + 0.078 * height (cm); and for CT muscle, 6.72 + 0.17 * height (cm) (Table S1a,b).^21^ The difference between measured LT (mean of triplicate) and the size‐related predicted latency time for both thoracic and pelvic limbs, respectively, was denominated ΔLT and used for comparison between recordings in the longitudinal study (Table S1a,b).

### 2.4 Data analysis

Data analysis was performed using R 4.3.1 (2023‐06‐16, R Foundation for Statistical Computing, Vienna, Austria).

Data was analysed to generate descriptive statistics. Univariable logistic regression was performed to test the associations of ΔLT and Knuckling grade in May versus October. Results with a *p*‐value <0.05 were considered statistically significant.

Cutoff values for increased ΔLT (delayed nerve signal) compared with expected values for ECR and CT muscles respectively were defined as mean ΔLT + 2SD from the measured values (*n* = 16) from May and October in horses (*n* = 4) without neurological signs or signs of lameness at any time point.

## 3 RESULTS

### 3.1 Clinical and neurological examinations

The general clinical examination, mental status, cranial nerves, and spinal reflexes were within normal limits in all examined horses both in May and October. None of the horses showed muscle atrophy. During the neurological examination in May, 14 horses showed neurological deficits, including 4 horses with a tendency to knuckle and 10 horses that displayed full knuckling (Table S1a,b). These horses are termed ‘affected’ by AEP in the remainder of the text. One horse knuckled in both thoracic and pelvic limbs (horse no. 14), and the rest only in the pelvic limbs. Knuckling was bilateral in most horses; in some cases, the impairment was slightly asymmetric. Clinical signs were most easily provoked when horses were circled, asked to back up, or when they were distracted or stressed. According to the personnel and the owners, most of the affected horses in May 2016 had been showing more pronounced signs earlier, at the beginning of the outbreak.

Six horses were deemed neurologically sound at the May examination, neither displaying knuckling nor a tendency to knuckle (grade 0; termed ‘unaffected’). These six horses had no history of knuckling. However, two of these horses showed right hindlimb lameness (lameness grade 2–3/5) at examination (horse no. 5 and 6; Table S1a,b).

After the first examination in May, all 14 affected horses were first rested from training, with daily turnouts in a small paddock. All except the most severely affected (horse no. 14) were then gradually put back to training, but 10 of the affected horses were reported to still show intermittent knuckling and not performing at pre‐outbreak levels in October. One of them (horse 7, North Swedish Draught) had shown worse fetlock instability (more frequent tendency for knuckling) than in May, but still scored grade 1.

At the follow‐up examination in October, knuckling grades had decreased in 13 out of 14 earlier affected (Table S1a,b). Four of 14 earlier knuckling and 6/6 earlier non‐knuckling horses were now classified as neurologically sound (grade 0). None of the four recovered horses had shown a relapse, that is, no worsening of the clinical signs once knuckling had ceased. Mean knuckling grades improved significantly between May and October (*p* = 0.04).

The 10 horses that still showed neurologic gait abnormalities in October mainly displayed mild bilateral metatarsophalangeal extensor dysfunction with a tendency to knuckle (grade 1) in the pelvic limbs together with mild dysmetria and spasticity (Tables 1 and S1a,b). Horse no. 14, which was the most severely affected in May, was not back in work and still showed grade 2 knuckling, albeit now only in the pelvic limbs.

### 3.2 Transcranial magnetic stimulation

**FIGURE 3:**
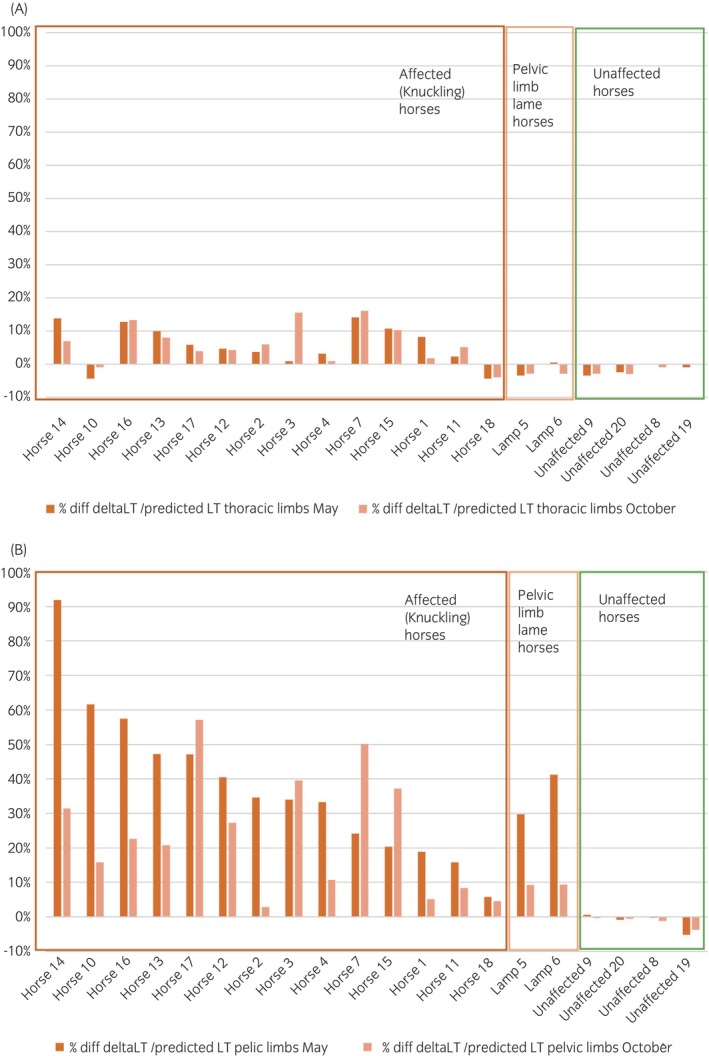
(A) Thoracic limbs: Relative delay (increase) in mean latency time from predicted value for transcranial magnetic motor evoked potentials in extensor carpi radialis muscle, in 20 horses from the premises with acquired equine polyneuropathy, examined in May and October 2016. Neurologically affected (knuckling) horses (*n* = 14), pelvic limb lame horses (*n* = 2), and unaffected horses (without clinical signs) (*n* = 4). Knuckling horses arranged in order from worst to least affected LT in pelvic limbs in May. Dark bar, May; light bar, October. (B) Pelvic limbs: Relative delay (increase) in mean latency time from predicted value for transcranial magnetic motor evoked potentials in cranial tibial muscles, in 20 horses from the premises with acquired equine polyneuropathy, examined in May and October 2016. Neurologically affected (knuckling) horses (*n* = 14), pelvic limb lame horses (*n* = 2), and unaffected horses (without clinical signs) (*n* = 4). Knuckling horses arranged in order from worst to least affected LT in pelvic limbs in May. Dark bar, May; light bar, October.

Mean values for LT and ΔLT for each horse (combined left and right side) after TMS are shown in Table S1a,b. The relative increase in mean LT from the predicted value for transcranial magnetic motor evoked potentials in neurologically affected horses, horses with pelvic limb lameness, and horses with no clinical signs are shown in Figure 3A,B. The onset latencies in the four clinically healthy horses measured both in May and October were within 95% prediction intervals presented by Nollet et al.,^21^ with mean ΔLT −0.35 ms (range, −0.7 to 0 ms; SD = 0.26; *n* = 8) for the ECR muscles in the thoracic limb, and − 0.5 ms (range, −1.8 to 0.2 ms; SD = 0.64; *n* = 8) for the CT muscles in the pelvic limb. The cutoff for increased ΔLT was set at mean + 2 SD of the healthy control group: >0.2 ms for thoracic limbs and >0.8 ms for pelvic limbs.

The two neurologically normal but lame horses (horses no. 5 and 6) were excluded from the calculations of cutoff values because they were not clinically sound. The ΔLTs were within reference in thoracic limbs and left pelvic limbs for both these horses but increased in their right pelvic limbs, worse in May than in October: horse no. 5, 21.8 ms (+65% above predicted value) in May and 6.5 ms (+19%) in October, and horse no. 6, 27.2 ms (+82%) in May and 6.1 ms (+18%) in October.

All affected horses (*n* = 14) showed delayed TMS responses to their CT muscles in the pelvic limbs (ΔLT exceeding the cutoff 0.8 ms) in both May and October, with mean ΔLT 12.95 ms in May (range 1.9–29.6 ms; SD 1.23; *n* = 14) and 8.1 ms in October (range 1–18.9 ms; SD 1.21; *n* = 14) (Table S1a,b). The mean difference from predicted LT in pelvic limbs in knuckling horses was +38% (range, 6%–92%) in May and +24% (range, 3–57%) in October (*p* = 0.062; Figure 3). In total, 10 out of the 14 originally knuckling horses improved their ΔLT in pelvic limbs between May and October, and improved clinical knuckling scores were observed in 13 of 14 affected horses.

However, in four horses (no 3, 7, 15 and 17), the ΔLTs in pelvic limbs had increased in October compared with May, despite their knuckling being clinically graded better in three and the same grade in one (Table S1b). Horses 7 (grade 1) and 17 (grade 0) were now the two horses with the most delayed TMS responses, +47 and +57% (ΔLT 16.4 and 18.9 ms) (Figure 3A,B).

Delayed TMS responses to the ECR muscles in the thoracic limbs (ΔLT exceeding the cutoff 0.2 ms) were observed in 11 out of the 14 horses in the affected group (79%) both in May and October (Figure 3B). Mean ΔLT in the thoracic limbs of the affected group was 1.24 ms in May (range, −0.9 to 2.9 ms; SD 1.23; *n* = 14) and 1.32 ms in October (range, −0.8 to 3.3 ms; SD 1.21; *n* = 14) (Table S1a). The mean difference from predicted LT in thoracic limbs in the affected horses (*n* = 14) was +6% (−4% to 14%) in May and +6% (−4% to 16%) in October.

### 3.3 Comparison between knuckling score and TMS

**FIGURE 4:**
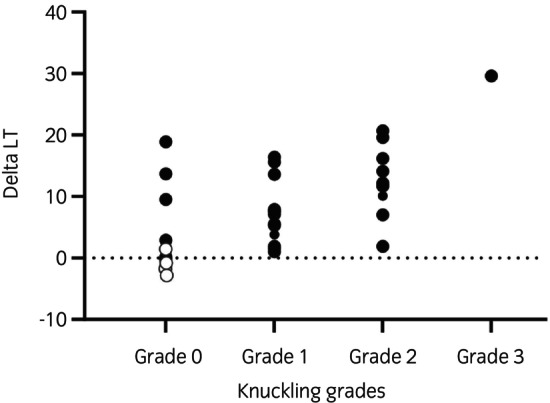
Clinical knuckling grades and the delta‐LT (ms) of pelvic limbs in the observations in May and October (*n* = 36) in horses that were or had been knuckling (*n* = 28 observations, filled circles) and unaffected horses (*n* = 8 observations, empty circles).

Knuckling grading scores and TMS results in pelvic limbs in the 36 observations of neurologically affected horses and unaffected horses (with no clinical signs) investigated in May and October are graphically presented in Figure 4. In neurologically unaffected (grade 0) horses, the mean ΔLT in pelvic limbs was 3.6 ms (range, −1.8 to 13.7 ms, *n* = 17). Grade 0 horses included both the horses (*n* = 4) that were unaffected at the start of the trial and horses that showed neurological signs in May but were found normal in October. All the horses that had recovered clinically still had delayed nerve conduction (*n* = 8, mean 24% increase in ΔLT for pelvic limbs, range, 8%–37%, Figure 4).

The ΔLT tended to be higher in horses with more severe signs of knuckling, but there was overlap in ΔLT between the different knuckling grades (Figure 4). Horses with a tendency for knuckling (grade 1) had a mean ΔLT 8.8 ms (range, 1.5–18.9 ms, *n* = 12); horses with knuckling grade 2 had a mean ΔLT 12.5 ms (range, 1.9–20.7 ms, *n* = 10) and one horse with knuckling grade 3 had ΔLT 29.6. In relative terms, there was a mean 26% (5%–57%) increase in ΔLT of pelvic limbs at grade 1 knuckling and a mean 36% increase (6%–62%) in horses with grade 2.

The most severely knuckling horse (horse no. 14, grade 3) also displayed the most delayed nerve conduction signals. LT in the pelvic limbs was 61.8 ms, ΔLT 29.6 ms, which is a 92% increase from normal (Table S1a,b; Figure 4). She was the only horse that also knuckled in the thoracic limbs.

## 4 DISCUSSION

This study demonstrates, for the first time, that AEP affects the conduction of the descending motor pathways of the nervous system. At first examination, the mean latency time difference (ΔLT) from normalised reference values^21^ in the pelvic limbs of neurologically affected horses was +12.95 ms, decreasing to +8.1 ms 5 months later. These values corresponded to mean increases of 38% and 24% compared with the study's cutoff values. Increased latency times were also observed in the thoracic limbs in several cases. To complement comparisons with reference values from Nollet et al.,^21^ a numerical cutoff specific to our study population was defined, >0.2 ms for thoracic limbs and >0.8 ms for pelvic limbs, offering a framework to evaluate percentage increases in LT. This approach provided context within the studied population and allows comparisons with healthy and affected horses, as well as results from other studies, like Nollet et al.,^21^ where we calculate that 95% prediction intervals for latency times based on withers height correspond to thresholds of ±4.82 ms for cranial tibial muscles and ±2.61 ms for extensor carpi radialis muscles from predicted LT. Notably, while alternative cutoffs might affect sensitivity and specificity, relative differences between measurements would remain consistent. Using Nollet's thresholds, 13/14 neurologically affected had increased latency times in pelvic limbs in May (93%), and 9/14 (64%) in October, and for thoracic limbs, 3/14 (21%) both in May and October.

This finding adds to the previously established involvement of sensory nerve fibres, specifically the decay of sensory Ia/Ib afferents.^8^ We have also documented, for the first time, the utility of TMS measurements to detect and quantify electrofunctional impairment in horses affected by AEP. Additionally, the delays in LT mainly corresponded to the degree of observed clinical signs. Full control of joint movements and stability depends upon both normal sensory input and normal motor activity, so knuckling in AEP could be a neurological sign of dysfunction in either sensory, motor, or both groups of nerve fibres. There is strong evidence for variable degrees of Schwann cell damage and secondary axonopathy of peripheral nerves.^8^ In cases of extensive axonopathy, recumbency would be expected, whereas nerves primarily affected by Schwannopathy exhibit prolonged conduction times due to the malfunctioning of saltatory conduction in myelinated nerve fibres. However, whereas sensory nerve fibre involvement was demonstrated in previous studies of AEP, histopathology alone cannot determine whether motor nerve fibres are also compromised in AEP, due to the lack of pure motor peripheral nerves in horses.^22^, ^23^ Pathohistological examination showed a large fibre neuropathy in AEP affected horses, pointing to involvement of either sensory Ia/Ib afferents or motor Aα‐efferents.^8^, ^9^ Histopathological examination showed that the dorsal nerve roots (purely sensory) were more affected than the ventral motor roots (that also may contain sensory fibres) in the spinal nerve roots of AEP horses.^8^, ^24^, ^25^ Ancillary tests for structural evaluation of peripheral motor pathway encompass the inspection of terminal intramuscular nerve endings and neuromuscular end plates, either by use of special markers (e.g., acetylcholine transporters and receptors) or by electron microscopy (endplates only). As these techniques provide a very limited insight on very high resolution, they only allow for qualitative assessment.

Quantitation and functional classification of lower motor neuron (LMN) abnormalities could be based on motor nerve conduction studies (MNCS). In horses, MNCS of specific peripheral nerves can only be performed under general anaesthesia or deep sedation and are therefore not easily performed in cases of AEP. Moreover, MNCS is not feasible in the distal part of the limbs due to equine nerve‐muscle anatomy, and as F wave and H reflex evaluations have not yet been reliably established in horses, MNCS is unlikely to pick up the functionality of proximal nerves and nerve roots.

Unfortunately, neither TMS nor the clinical signs in AEP can distinguish between UMN or LMN lesions. The pathomorphology in AEP horses is consistent with a demyelinating polyneuropathy of peripheral nerve fibres, leading us to expect that the latency increase demonstrated by TMS is at least partly due to impaired neuronal conduction in the peripheral motor pathways. Nevertheless, the delay in LT cannot exclude additional dysfunction of the CNS motor pathways. To determine the full spectrum of pathology in this disease, histopathology of neuromuscular end plates in addition to more extensive central nervous system examinations is necessary.

Histopathological studies have shown consistent demyelinating lesions across all peripheral nerves in AEP and an even distribution between left and right,^8^ findings that are supported by this study. In addition, clinical signs indicate that AEP primarily affects the pelvic limbs initially, progressing in some cases to the thoracic limbs. TMS measurements conducted in this study strongly support this observation, revealing significantly delayed motor nerve signal conduction to the pelvic limbs (mean ΔLT 12.95 ms) compared with the thoracic limbs (mean ΔLT 1.72 ms). The severity of clinical signs is largely linked to the extent of these delays. Despite similar neuropathological lesions in all nerves, these results suggest that muscles dependent on longer motor pathways, such as those in the pelvic limbs, are inherently more susceptible to dysfunction due to their greater length and resulting longer conduction delays. Longer nerves consisting of numerous individual fibres provide more overall surface for a toxicant to damage. Delay may also be exacerbated by the summation effect. The sensory and motor nerve involvement both add to the mismatch of ascending and descending nerve signals, which likely contributes to the observed clinical manifestations.

None of the affected horses in this study showed muscle wasting at this stage of disease. This is consistent with the subtle and inconsistent evidence of myofibre atrophy in muscle biopsies previously procured from AEP horses.^5^, ^8^ Atrophy in demyelinating and Schwannopathic nerve diseases, as AEP, lacks denervation features until very late stages. In the same vein, one single case of AEP examined by EMG revealed no spontaneous activity typical for loss of motor axons.^5^ As the muscle therefore provides an incomplete insight into AEP associated nerve pathology and nerve biopsies are rarely performed, one might conclude that TMS with measurements of motor potential latency times could be a valuable and reproducible method in AEP to objectively assess the motor fibre dysfunction in the horses' gait deficits, including its improvement or worsening with time.^26^ We know from previous AEP outbreaks that the severity of clinical signs may differ considerably between affected horses on the same farms and across different time points in individual horses.^1^ In the current study, clinical improvement was mostly reflected by shorter onset latencies. Interestingly, at follow‐up during recovery, all four horses that previously exhibited knuckling and now were classified as grade 0 still showed increased latency times, indicating incomplete recovery and lingering subclinical impairment of the descending motor pathways. Thus, TMS seems to be a more sensitive method to detect and score AEP affected horses, including subclinical cases, compared with a single crude clinical and neurological score.

A subgroup of four horses (numbers 3, 7, 15, and 17) showed increased latency times in October compared with May, despite showing improved or stable clinical grading of knuckling. In previous convalescent AEP cases, we have observed that mild metatarsophalangeal extension dysfunction can fluctuate daily and may be difficult to detect during a short neurological exam. This underscores the value of repeated clinical observations by the owner as an important adjunct. Additionally, horses may develop compensatory mechanisms to place their limbs correctly despite neurological damage, or there may be temporal changes in the balance between sensory and motor function. Improvements in muscle strength or coordination may be attributed to better cortical or subcortical control rather than changes in peripheral conduction. Enhanced compensatory mechanisms in the motor cortex or spinal cord may mask deficits detected in motor latency tests. Motor latency times are strongly influenced by the integrity of the myelin sheath and axonal conduction velocity, and structural recovery may lag behind clinical functional recovery.^27^ Furthermore, histopathology of nerves from some recovered AEP horses has shown signs of inflammatory demyelinating neuropathy even years after clinical signs have resolved (unpublished data). Accordingly, the inclusion of six neurologically sound stablemates in the present study was justified, as subclinical AEP cases can occur at any time during an outbreak. Two of these horses were excluded from the non‐clinical group due to hindlimb lameness. Follow‐up revealed chronic lameness in both cases, and both horses were eventually euthanised. Delayed latency times to the affected limbs observed on TMS suggest neurological involvement beyond orthopaedic issues. While a neurological cause unrelated to AEP is plausible, investigating the exact cause of the lameness was beyond the scope of this study.

One weakness of this study of AEP was that examinations were only repeated once, for organisational reasons. A longitudinal study starting earlier in the disease, repeated at shorter intervals, and lasting for a couple of years would allow closer investigation of how LTs and ΔLT can vary over time with clinical progression of AEP, and what the LT delays are when the clinical deficits are peaking as well as in subclinical disease. Moreover, transcranial electrical stimulation (TES) may serve as a more robust technique in further studies, comprising better reproducibility and more properly defined cutoff values to discern between health and disease.^15^, ^28^

In conclusion, this study demonstrates for the first time that AEP involves the descending motor pathways, rather than being a disease exclusively affecting the sensory nervous system. Furthermore, the study highlights how TMS aids in objectively measuring motor pathway damage and monitoring improvement in horses affected by AEP. This technique may therefore be a suitable non‐invasive diagnostic tool for evaluating the clinical course of AEP and identifying subclinically or minimally affected horses.

## FUNDING INFORMATION

This study was partly funded by The Swedish Association for the Protection of Animals (Svenska Djurskyddsföreningen). Most costs were covered by Ludwig‐Maximilians‐University, Munich, Germany.

## CONFLICT OF INTEREST STATEMENT

The authors have declared no conflicting interests.

## AUTHOR CONTRIBUTIONS

**Anna May:** Conceptualization; investigation; writing – original draft; visualization; writing – review and editing; formal analysis; project administration; data curation. **Siv Hanche‐Olsen:** Investigation; writing – review and editing; resources. **Lutz S. Goehring:** Conceptualization; funding acquisition; writing – review and editing; methodology; project administration; resources; supervision. **Kaspar Matiasek:** Writing – review and editing; conceptualization; data curation. **Karin Hultin Jäderlund:** Investigation; writing – review and editing; methodology. **Yury Zablotski:** Methodology; software. **Gittan Gröndahl:** Conceptualization; investigation; writing – review and editing; writing – original draft; project administration; supervision; resources; data curation.

## DATA INTEGRITY STATEMENT

Anna May and Gittan Gröndahl had full access to all the data in the study and take responsibility for the integrity of the data and the accuracy of data analysis.

## ETHICAL ANIMAL RESEARCH

Research ethics committee oversight not currently required by this journal: procedures were performed as part of clinical investigations.

## INFORMED CONSENT

Owners gave consent for their animals' inclusion in the study.

## ANTIMICROBIAL STEWARDSHIP POLICY

Not applicable.

## Supporting information


**Table S1.** Supporting Information.

## Data Availability

The data that support the findings of this study are openly available in Open Data LMU at https://doi.org/10.5282/ubm/data.592 from the date of publication.
