# Supplementary material for: Motor pathway evaluation by transcranial magnetic stimulation in Swedish horses with acquired equine polyneuropathy
Source: Equine Vet J. 2025 Apr 21;58(1):115–24. doi: 10.1111/evj.14506 (PMC12699128; doi:10.1111/evj.14506)
Supplement: Supplementary file 1 — Table S1. Supporting Information. [file EVJ-58-115-s001.pdf]

Equine Veterinary Journal BEVA

Tables S1a and S1b: Results from transcranial magnetic stimulation, height measurements, and clinical grading of knuckling (generally only in pelvic limbs) in 20 horses from a premise with acquired equine polyneuropathy, examined in May and October 2016. Knuckling horses are arranged in order from worst to least affected in pelvic limbs. LT, latency time for magnetic motor evoked potentials in respectively extensor carpi radialis (ECR, Table S1a) and cranial tibial (CT, Table S1b) muscles, mean of triplicates from left and right side. Delta-LT, difference between predicted and recorded LT. K=knuckling, NK= non knuckling. Predicted latency time was based on withers height, according to Nollet et al.: ECR 8.55 + 0.078 x height (cm), CT 6.72 + 0.17 x height (cm).[18] Differences in results from May to October are highlighted in red for increased values and blue for decreased values.

LH=left hind, RH=right hind

a. Thoracic limbs, Extensor carpi radialis muscle (ECR)

			May 2016				October 2016			
**Horse no.**	**Withers height in cm**	**Predicted latency time (LT in ms)**	**LT (ms)**	**Delta-LT (ms)**	**Knuckling grade**		**LT (ms)**	**Delta-LT (ms)**	**Knuckling grade**	
**Knuckling horses (arranged from worst to least affected in pelvic limbs)**										
**14**	150	20.3	23.1	2.8	K	3	21.7	1.4	K	2
**10**	158	20.9	20.0	-0.9	K	2	20.7	-0.2	K	1
**16**	161	21.1	24.4	2.7	K	2	24.5	2.8	K	1
**13**	162	21.2	23.3	2.1	K	2	22.9	1.7	K	1
**17**	155	20.6	21.8	1.2	K	1	21.4	0.8	NK	0
**12**	165	21.4	22.4	1.0	K	2	22.3	0.9	NK	0
**2**	168	21.7	22.5	0.8	K	2	23.0	1.3	K	1
**3**	163	21.3	21.5	0.2	K	2	24.6	3.3	K	1
**4**	169	21.7	22.4	0.7	K	2	21.9	0.2	K	1
**7**	153	20.5	23.4	2.9	K	1	23.8	3.3	K	1
**15**	177	22.4	24.8	2.4	K	1	24.7	2.3	NK	0
**1**	172	22.0	23.8	1.8	K	2	22.4	0.4	K	1
**11**	165	21.4	21.9	0.5	K	1	22.5	1.1	NK	0
**18**	155	20.6	19.7	-0.9	K	2	19.8	-0.8	K	1
**Pelvic limb lame horses**										
**5**	158	20.9	20.2	-0.7	NK	0	20.3	-0.6	NK	0
**6**	156	20.7	20.8	0.1	NK	0	20.1	-0.6	NK	0
**Unaffected horses**										
**8**	165	21.4	21.4	0	NK	0	21.2	-0.2	NK	0
**9**	156	20.7	20.0	-0.7	NK	0	20.1	-0.6	NK	0
**19**	164	21.3	21.1	-0.2	NK	0	21.3	0	NK	0
**20**	155	20.6	20.1	-0.5	NK	0	20.0	-0.6	NK	0

b. Pelvic limbs, Tibial cranial muscle (CT)										
			May 2016				October 2016			
Horse no.	Withers height in cm	Predicted latency time (LT in ms)	LT (ms)	Delta-LT (ms)	Knuckling grade		LT (ms)	Delta-LT (ms)	Knuckling grade	
Knuckling horses (arranged from worst to least affected in pelvic limbs)										
**14**	150	32.2	61.8	29.6	K	3	42.3	10.1	K	2
**10**	158	33.6	54.3	20.7	K	2	38.9	5.3	K	1
**16**	161	34.1	53.7	19.6	K	2	41.8	7.7	K	1
**13**	162	34.3	50.5	16.2	K	2	41.4	7.1	K	1
**17**	155	33.1	48.7	15.6	K	1	52.0	18.9	NK	0
**12**	165	34.8	48.9	14.1	K	2	44.3	9.5	NK	0
**2**	168	35.3	47.5	12.2	K	2	45.3	1.0	K	0
**3**	163	34.4	46.1	11.7	K	2	48.0	13.6	K	1
**4**	169	35.5	47.3	11.8	K	2	39.3	3.8	K	1
**7**	153	32.7	40.6	7.9	K	1	49.1	16.4	K	1
**15**	177	36.8	44.3	7.5	K	1	50.5	13.7	NK	0
**1**	172	37.1	44.1	7.0	K	2	39.0	1.9	K	1
**11**	165	34.8	40.3	5.5	K	1	37.7	2.9	NK	0
**18**	155	33.1	35.0	1.9	K	2	34.6	1.5	K	1
Pelvic limb lame horses										
**5**	158	33.6	44.6, LH: 33.7, RH: 55.4	10.0, LH: 0.1, RH: 21.8	NK, lame RH	0	36.7, LH: 33.2, RH: 40.1	3.1, LH: -0.4, RH: 6.5	NK	0
**6**	156	33.2	46.9, LH: 33.4, RH: 60.4	13.7, LH: 0.2, RH: 27.2	NK, lame RH	0	34.8, LH: 30.3, RH: 39.3	3.1, LH: -2.9, RH: 6.1	NK	0
Unaffected horses										
**9**	156	33.2	33.4	0.2	NK	0	33.1	-0.1	NK	0
**20**	155	33.1	32.8	-0.3	NK	0	32.9	-0.2	NK	0
**8**	165	34.8	34.7	-0.1	NK	0	34.4	-0.4	NK	0
**19**	164	34.6	32.8	-1.8	NK	0	33.3	-1.3	NK	0
